# Cyclin-dependent Kinase 9 as a Potential Target for Anti-TNF-resistant Inflammatory Bowel Disease

**DOI:** 10.1016/j.jcmgh.2022.05.011

**Published:** 2022-06-01

**Authors:** Omer S. Omer, Arnulf Hertweck, Luke B. Roberts, Jonathan W. Lo, Jennie N. Clough, Ian Jackson, Eirini D. Pantazi, Peter M. Irving, Tom T. MacDonald, Polychronis Pavlidis, Richard G. Jenner, Graham M. Lord

**Affiliations:** 1 School of Immunology and Microbial Sciences, King's College London, London, UK; 2 National Institute for Health Research Biomedical Research Centre at Guy’s and St Thomas’ NHS Foundation Trust and King’s College, London, UK; 3 UCL Cancer Institute and CRUK City of London Centre, University College London, London, UK; 4 Division of Digestive Diseases, Faculty of Medicine, Imperial College, London, UK; 5 Kennedy Institute of Rheumatology, University of Oxford, Oxford, UK; 6 Inflammatory Bowel Disease Unit, Department of Gastroenterology, Guy's and St Thomas' NHS Foundation Trust, London, UK; 7 Centre for Immunobiology, Blizard Institute, Barts and the London School of Medicine and Dentistry, Queen Mary University of London, London, UK; 8 Faculty of Biology, Medicine and Health, University of Manchester, Manchester, UK

**Keywords:** CDK9, Crohn’s Disease, IBD, Inflammation, Ulcerative Colitis, CD, Crohn’s disease, CKD9, cyclin-dependent kinase 9, CKD9i, CDK9 inhibitor, CTD, carboxyl-terminal domain, DMSO, dimethyl sulfoxide, ELISA, enzyme-linked immunosorbent assay, FACS, fluorescence-activated cell sorting, FCS, fetal calf serum, FDR, false discovery rate, GSEA, gene set enrichment analysis, GSVA, gene set variation analysis, IBD, inflammatory bowel disease, IFN, interferon, IL, interleukin, IPA, ingenuity pathway analysis, LP, lamina propria, LPMCs, lamina propria mononuclear cells, MACS, magnetic-activated cell separation, PB, peripheral blood, PMA, phorbol 12-myristate 13-acetate, PMA/I, PMA and ionomycin, PUCAI, Paediatric UC Activity Index, RNA pol II, RNA polymerase II, RT-qPCR, reverse transcription quantitative polymerase chain reaction, S2P, phospho-serine 2, TCT, T cell transfer, Th, T helper, TNF-α, tumor necrosis factor-alpha, TSSs, transcription start sites, UC, ulcerative colitis, WT, wild-type

## Abstract

**Background & Aims:**

Resistance to single cytokine blockade, namely anti-tumor necrosis factor (TNF) therapy, is a growing concern for patients with inflammatory bowel disease (IBD). The transcription factor T-bet is a critical regulator of intestinal homeostasis, is genetically linked to mucosal inflammation and controls the expression of multiples genes such as the pro-inflammatory cytokines interferon (IFN)-γ and TNF. Inhibiting T-bet may therefore offer a more attractive prospect for treating IBD but remains challenging to target therapeutically. In this study, we evaluate the effect of targeting the transactivation function of T-bet using inhibitors of P-TEFb (CDK9-cyclin T), a transcriptional elongation factor downstream of T-bet.

**Methods:**

Using an adaptive immune-mediated colitis model, human colonic lymphocytes from patients with IBD and multiple large clinical datasets, we investigate the effect of cyclin-dependent kinase 9 (CDK9) inhibitors on cytokine production and gene expression in colonic CD4^+^ T cells and link these genetic modules to clinical response in patients with IBD.

**Results:**

Systemic CDK9 inhibition led to histological improvement of immune-mediated colitis and was associated with targeted suppression of colonic CD4^+^ T cell-derived IFN-γ and IL-17A. In colonic lymphocytes from patients with IBD, CDK9 inhibition potently repressed genes responsible for pro-inflammatory signalling, and in particular genes regulated by T-bet. Remarkably, CDK9 inhibition targeted genes that were highly expressed in anti-TNF resistant IBD and that predicted non-response to anti-TNF therapy.

**Conclusion:**

Collectively, our findings reveal CDK9 as a potential target for anti-TNF-resistant IBD, which has the potential for rapid translation to the clinic.

## Graphical abstract

**Figure:**
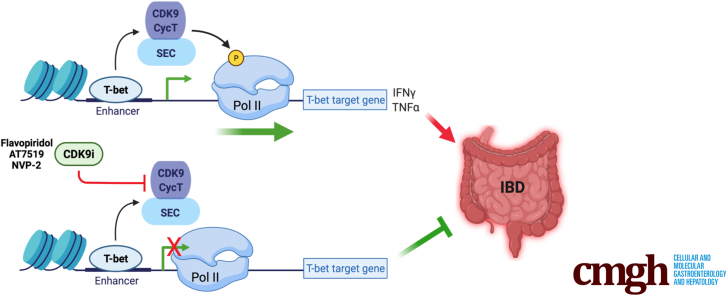

**Summary**

Cyclin-dependent kinase 9 inhibition represents a potential mechanism for repressing dysregulated immune pathways implicated in anti-tumor necrosis factor-resistant inflammatory bowel disease.

Inflammatory bowel disease (IBD) primarily consists of 2 conditions, ulcerative colitis (UC) and Crohn's disease (CD). Gastrointestinal inflammation in UC is confined to the colonic mucosa, whereas in CD, transmural inflammation can affect any part of the gut from mouth to anus.^1^^,^^2^ Aberrant immune cell activation can lead to tissue destruction, resulting in complications such as fulminant colitis, gastrointestinal structuring, and fistulae formation. These devastating complications, especially in younger patients, cannot be underestimated.

The therapeutic landscape of IBD has evolved over the past few decades with the introduction of monoclonal antibodies against tumor necrosis factor-alpha (TNF-α), integrin-α_4_β_7_, and interleukin (IL)-12/23 p40.^3^ Nevertheless, significant problems remain. These include primary unresponsiveness to anti-TNF agents, waning of responsiveness with time, and lack of biomarkers predicting responsiveness to a particular therapy.^4^^,^^5^

Targeting individual pro-inflammatory molecules in IBD has yielded mixed results. Positive clinical outcomes have been reported with anti-TNF (infliximab, adalimumab, golimumab) and anti-IL-12/23 p40 (ustekinumab) therapy.^6^, ^7^, ^8^, ^9^ By contrast, monoclonal antibodies against IL-17A (secukinumab) and interferon (IFN)-γ (fontolizumab) have failed to achieve primary endpoints in clinical trials and, in some cases, were associated with worsening disease activity.^10^^,^^11^ Targeting specific transcription factors may offer a more attractive prospect for treating IBD, as they lie upstream of effector cytokines and additionally regulate hundreds of related immune response genes acting in the same pathway.

The lineage-determining transcription factor, T-bet (encoded by *TBX21*), plays a critical role in maintaining gastrointestinal immune homeostasis, striking a balance between host defence and immune tolerance.^12^ T-bet regulates T helper (Th) cell development, promoting Th1 cell differentiation whilst suppressing Th2 and Th17 lineage commitment.^13^ In Th1 cells, T-bet directly activates *IFNG* and *TNF*, ensuring coordinated production of the effector cytokines encoded by these genes and promoting a cell-mediated immune response against pathogens.^14^ Single nucleotide polymorphisms at T-bet binding sites can alter T-bet binding and are associated with mucosal autoimmune diseases such as IBD and celiac disease.^15^

There is a wealth of data from experimental colitis models and human IBD tissue supporting a significant role for Th1 responses in mediating intestinal inflammation. Experimental colitis secondary to adoptive transfer of naïve T cells, 2,4,6-trinitrobenzene sulfonic acid administration, and spontaneous colitis in IL-10 deficiency mouse models are all associated with increased T-bet expression in lamina propria (LP) T cells.^16^ By contrast, *Tbx21* and *Ifng* knockout models are protected against adoptive transfer colitis.^16^^,^^17^ In humans, increased T-bet expression was observed in LP T cells from patients with intestinal inflammation, most notably those with CD.^16^^,^^18^ T cells isolated from areas of active CD also produced high levels of IFN-γ compared with healthy controls, and T cell receptor activation induced T-bet expression.^18^, ^19^, ^20^ Anti-inflammatory therapies, such as glucocorticoids, which are somewhat effective in the management of exacerbations of IBD, have been shown to inhibit T-bet function.^21^ Taken together, these findings suggest that inhibiting T-bet activity in CD4^+^ T cells may have therapeutic potential in IBD.

Despite the appeal of inhibiting T-bet, transcription factors are difficult to target due to their lack of enzymatic activity suitable for chemical intervention.^22^ Dissection of the molecular mechanisms employed by T-bet to regulate Th1 gene expression has identified 2,4,6-trinitrobenzene sulfonic acid as a potential surrogate target of T-bet transcriptional activity.^23^ T-bet acts through enhancer regions to allow the recruitment of Mediator and the transcriptional elongation factor P-TEFb to Th1 genes.^23^ P-TEFb consists of a catalytic subunit, cyclin-dependent kinase 9 (CDK9), and either cyclin T1 or cyclin T2.^24^ CDK9 is responsible for activating transcriptional elongation by phosphorylating DRB sensitivity-inducing factor, negative elongation factor, and serine 2 on the carboxyl-terminal domain (CTD) of RNA polymerase II (RNA pol II).^25^ We previously demonstrated that the CDK9 inhibitor (CDK9i) flavopiridol inhibited expression of Th1 genes and abrogated Th1-mediated autoimmune uveitis.^23^ Flavopiridol has only limited specificity for CDK9, but other more potent and specific CDK9i have been developed, including AT7519 (IC_50_ 47 nM)^26^^,^^27^ and NVP-2 (IC_50_ 0.5 nM).^28^

The demonstration that CDK9 inhibition represses Th1 gene expression has raised the possibility of ameliorating intestinal inflammation via blockade of P-TEFb-mediated transcriptional elongation. In this study, we demonstrate that CDK9 inhibition supresses inflammatory cytokine production in an adoptive transfer colitis model and in colonic tissue from patients with IBD. Using transcriptomic data, we show that CDK9 inhibition represses genes associated with pro-inflammatory signalling. CDK9i-repressed genes were highly expressed in patients with anti-TNF resistant UC and colonic CD and were predictive of response to anti-TNF therapy. Together, these data identify CDK9 as a potential therapeutic target in IBD and defines a CDK9-dependent transcriptional signature as a biomarker predictive of anti-TNF resistance.

## Results

### CDK9 Inhibition Suppresses IFN-γ and TNF-α Production by CD4^+^ T Cells

**Figure 1:**
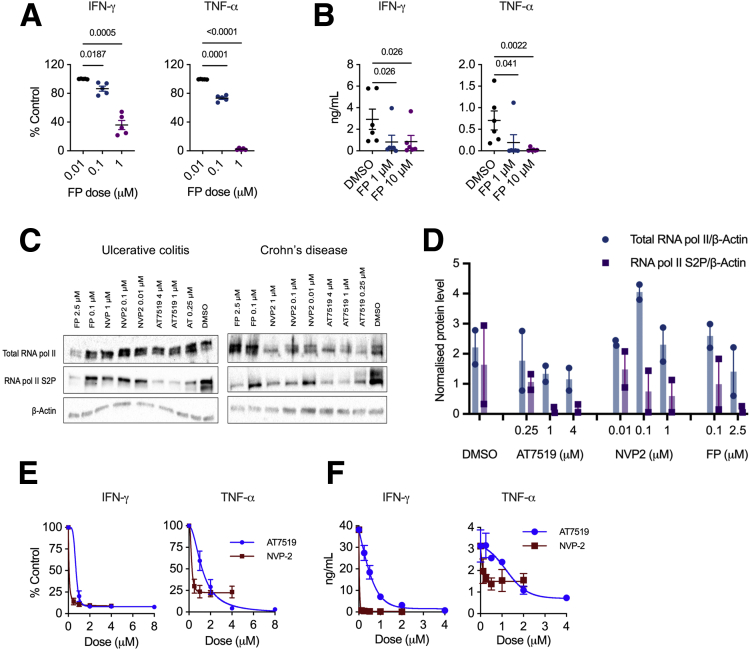
**CDK9 inhibition suppresses IFN-γ and TNF-α production in murine and human CD4**^**+**^**T cells.***A*, Proportions of murine CD4^+^ Th1 cells positive for intracellular cytokines following CDK9 inhibition with flavopiridol (FP) relative to untreated (DMSO control). Mean ± standard error of the mean (SEM); n = 5. Paired Student *t* test. *B*, Cytokine concentration in the supernatants of TCT colitis explants following 27-hour treatment with FP. Mean ± SEM; IFN-γ assays, n = 4; TNF-α assays, n = 6. Mann-Whitney *U* test. *C*, Immunoblots for RNA pol II S2P and total RNA pol II in PB CD4^+^ T cells purified from a patient with UC and a patient with CD and re-stimulated for 3 hours in the presence of DMSO, FP, NVP-2, or AT7519. *D*, RNA pol II S2P and total RNA pol II levels, normalized to β-actin. Mean ± SEM; n = 2. *E*, Proportions of human patient with IBD PB CD4^+^ T cells positive for intracellular cytokines following 3-hour treatment with AT7519 or NVP-2 relative to untreated (DMSO control). Mean ± SEM; n = 4. *F*, IFN-γ and TNF-α concentration in supernatant from patients with IBD PB CD4^+^ T cells restimulated for 16 hours in the presence of AT7519 or NVP-2. Mean ± SEM; n = 2.

To determine the effect of CDK9 inhibition on IFN-γ and TNF-α production, wild-type (WT) naïve murine CD4^+^ T cells were activated with anti-CD3 and anti-CD28 in the presence of IL-12 and anti-IL-4 to induce Th1 cell differentiation. Th1 cells were then restimulated with phorbol 12-myristate 13-acetate (PMA) and ionomycin (PMA/I) in the presence of flavopiridol for 4 hours. Intracellular cytokine staining demonstrated dose-dependent suppression of IFN-γ and TNF-α by flavopiridol (Figure 1, *A*). Next, we investigated whether CDK9 inhibition could directly suppress cytokine production in colonic explants from mice with established T cell transfer (TCT) colitis. Explants were cultured with flavopiridol for 27 hours, and cytokine concentration was quantified using enzyme-linked immunosorbent assay (ELISA). IFN-γ and TNF-α concentrations were significantly reduced in the supernatant of explants following CDK9 inhibition (Figure 1, *B*).

To investigate the relevance of these findings to human disease, we first assessed the activity of CDK9 inhibition in peripheral blood (PB) CD4^+^ T cells from patients with IBD. Immunoblotting was utilized to quantify RNA pol II phospho-serine 2 (S2P), which is catalyzed by P-TEFb. PB CD4^+^ T cells were activated using anti-CD3 and anti-CD28 for 72 hours then rested for 48 hours. Cells were then restimulated with PMA/I for 3 hours in the presence of the CDK9i flavopiridol, AT7519, or NVP-2. Analysis revealed a dose-dependent reduction in RNA pol II S2P levels following CDK9 inhibition (Figures 1, *C–D*).

Using the same experimental protocol, the more selective compounds, AT7519 and NVP-2, were utilized to evaluate the effect of CDK9 inhibition on Th1 cytokine production in PB CD4^+^ T cells from patients with IBD. Flow cytometry revealed dose-dependent suppression of IFN-γ and TNF-α following 3 hours of CDK9 inhibition (Figure 1, *E*). Although both compounds had equal effects on IFN-γ, TNF-α inhibition saturated for NVP-2 but not for AT7519. We sought to validate these results using ELISA. CD4^+^ T cells were restimulated for 16 hours in the presence of NVP-2 or AT7519 and supernatant collected for cytokine quantification. IFN-γ and TNF-α concentrations were reduced following CDK9 inhibition, and dose-response characteristics of NVP-2 and AT7519 mirrored the findings observed by flow cytometry (Figure 1, *F*). We conclude that CDK9 inhibition supresses the production of Th1 cytokines in murine and human CD4^+^ T cells.

### CDK9 Inhibition is Associated With Histological Improvement of T Cell Transfer Colitis

**Figure 2:**
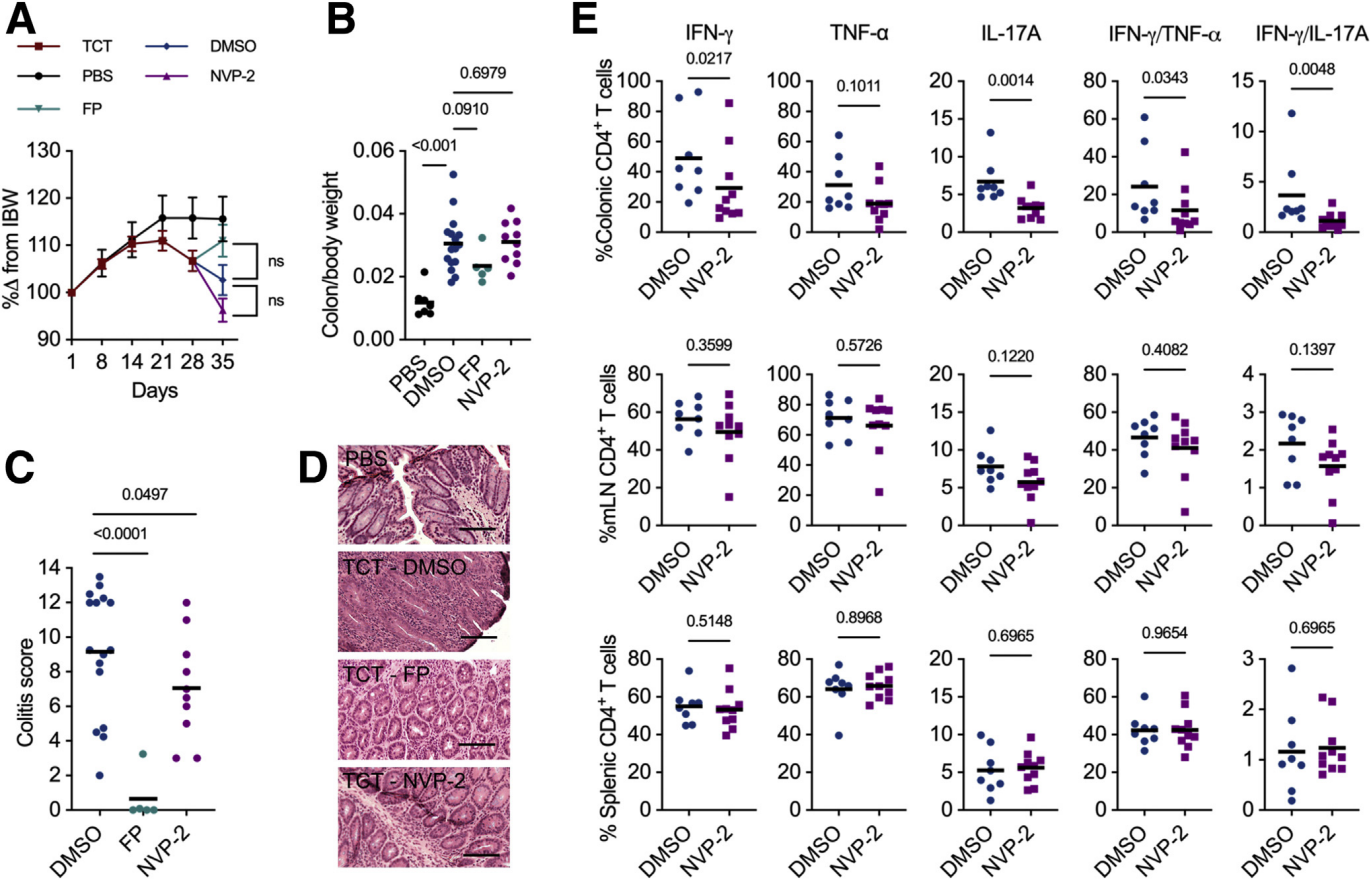
**CDK9 inhibition leads to histological improvement of TCT colitis.***A*, Percentage change from initial bodyweight (IBW) of mice following intraperitoneal injection of 0.5 ×10^6^ WT CD4^+^ CD25^-^ CD62L^+^ CD44^low^ T cells. Mean ± standard error of the mean; TCT colitis (n = 28), PBS (n = 5), 1% DMSO (n = 11), 3 mg/kg flavopiridol (FP, n = 5), 1 mg/kg NVP-2 (n = 10). Two-way analysis of variance. *B*, Normalized colon weights (colon weight [g] / bodyweight [g]) of control mice that received PBS instead of T cells (n = 7) compared with TCT mice following treatment with DMSO (n = 16), FP (n = 5), or NVP-2 (n = 10). Mann-Whitney *U* test. *C*, Histological colitis scores of TCT mice following treatment with DMSO (n = 16), FP (n = 5), or NVP-2 (n = 10). One-tailed Mann-Whitney *U* test. *D*, Representative colon micrographs for control mice (PBS) compared with DMSO-, FP-, or NVP-2-treated TCT mice (hematoxylin and eosin stained). Scale bars 250 μm. *E*, Proportions of CD4^+^ T cells positive for intracellular cytokines from colons, mesenteric lymph nodes (mLNs), or spleens of mice treated with 1 mg/kg NVP-2 (n = 10) or 1% DMSO (n = 8). Mann-Whitney *U* test.

Having established that CDK9 inhibition effectively suppresses IFN-γ and TNF-α production in CD4^+^ T cells, we reasoned that systemic delivery of CDK9i could abrogate experimental colitis. Adoptive transfer of naïve CD4^+^ T cells into mice that lack an adaptive immune system induces transmural colonic inflammation that histologically resembles CD.^29^ In this disease model, colitis is driven by the expansion of pathogenic Th1 and Th17 cells in the absence of regulatory T cells.^29^ Thus, TCT colitis represents an appropriate model to evaluate the response to CDK9 inhibition. Following the adoptive transfer of naïve CD4^+^ CD25^-^ CD62L^+^ CD44^low^ T cells into *Rag2*^*-/-*^ mice, colitis ensued by day 30, with mice suffering weight loss and diarrhea (Figure 2, *A*). After establishment of colitis, flavopiridol, NVP-2, or dimethyl sulfoxide (DMSO) were administered once daily by intraperitoneal injection, and mice were subsequently sacrificed on day 6 of treatment. There was no difference in weight loss or normalised colon weights between the CDK9i-treated mice and DMSO control (Figure 2, *A–B*). There was, however, an improvement in colitis scores in mice receiving systemic CDK9 inhibition, with the greatest effect observed with flavopiridol (Figure 2, *C–D*). Next, we sought to determine whether the histological improvement in colitis could be related to pro-inflammatory cytokine suppression. Mice receiving the highly selective CDK9i NVP-2 were used to test this concept. Flow cytometric analysis of CD4^+^ T cells isolated from mice treated with NVP-2 revealed a significant reduction in pro-inflammatory cytokine expression in colonic T cells, which was not observed in the mesenteric lymph nodes or spleen (Figure 2, *E*). Together, these findings suggest that systemic CDK9 inhibition targets aberrant effector cytokine production at the site of inflammation and can be used to attenuate adaptive immune-mediated colitis.

### CDK9 Inhibition in Colonic Lamina Propria Mononuclear Cells Leads to Transcriptional Repression of IFN-γ, but also the Th17 Cytokine, IL-17A

**Figure 3:**
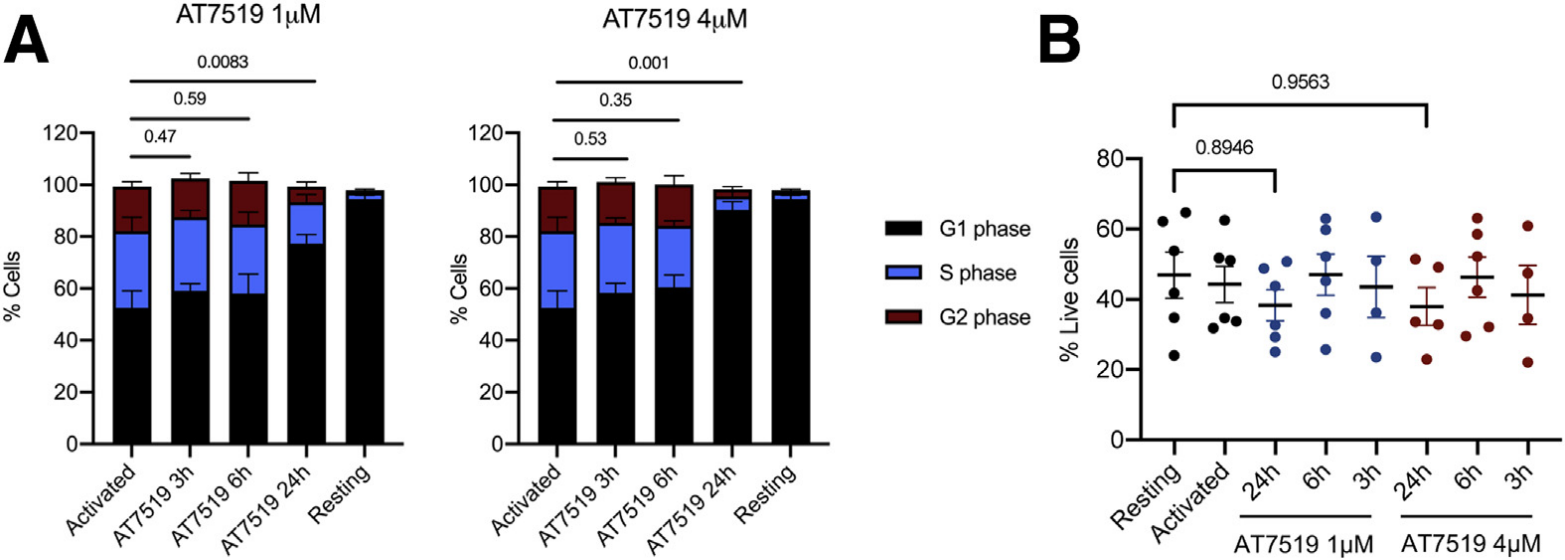
**Short treatments of CDK9 inhibition do not cause cell cycle arrest.***A*, Proportion of CD4^+^ LPMCs in G1, S, and G2 phase following activation with anti-CD3 and anti-CD28 for 48 hours and 3-hour, 6-hour, or 24-hour treatment with 1 or 4 mM AT7519. Activated control represents CD4^+^ LPMCs cultured with anti-CD3 and anti-CD28 alone for 48 hours. Resting control represents CD4^+^ LPMCs cultured without anti-CD3 and anti-CD28. Mean ± standard error of the mean; n = 6. Unpaired 2-tailed Student *t* test estimating significance of differences in % cells in G1. *B*, Proportion of live colonic LPMCs following 3 hours, 6 hours, and 24 hours of treatment with AT7519. Mean ± standard error of the mean, n = 6. Unpaired 2-tailed Student *t* test.

We next sought to establish the effect of CDK9 inhibition on colonic lamina propria mononuclear cells (LPMCs) from patients with IBD. We first wanted to confirm that any effects of the inhibitors on cytokine production were not caused by inhibition of other CDKs that regulate cell-cycle progression. Colonic biopsies were cultured on Cellfoam matrices, and LPMCs were harvested as previously described.^30^ LPMCs were activated and cultured with AT7519 for 3, 6, and 24 hours, and cell-cycle phases were assessed by flow cytometry. There was no difference in the proportion of cells in each phase of the cell cycle or cell viability following 3 or 6 hours of CDK9 inhibition (Figure 3, *A–B*), although there was evidence of G1 arrest after 24 hours of treatment (Figure 3, *A*). These findings show that short treatments with CDK9i do not cause cell-cycle arrest.

**Figure 4:**
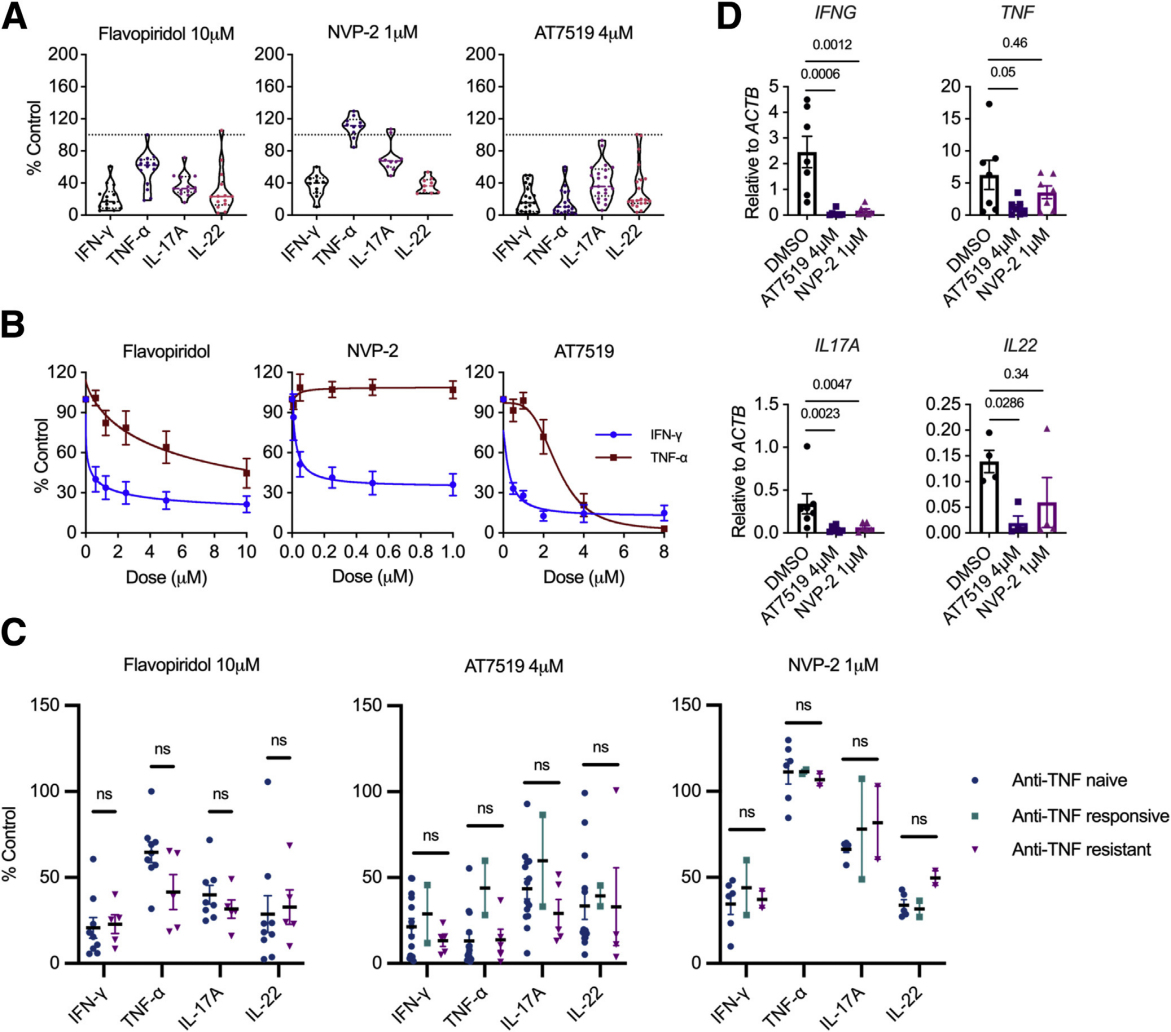
**CDK9 inhibition suppresses transcription of Th1 and Th17 cytokines.***A*, Proportions of CD4^+^ LPMCs positive for intracellular cytokines following 3-hour incubation with 10 μM flavopiridol (n = 14), 4 μM AT7519 (n = 21), or 1 μM NVP-2 (n = 10) relative to untreated (DMSO control). Median ± interquartile range. *B*, Dose-response curves for intracellular cytokines in CD4^+^ LPMCs following 3-hour treatment with flavopiridol (n = 5), AT7519 (n = 6), or NVP-2 (n = 5); Mean ± standard error of the mean. *C*, Proportions of CD4^+^ LPMCs positive for intracellular cytokines following 3-hour incubation with 10 μM flavopiridol (n = 14), 4 μM AT7519 (n = 21), or 1 μM NVP-2 (n = 10) relative to untreated (DMSO) control. Results stratified by patients on anti-TNF therapy with active disease (anti-TNF resistant; flavopiridol, n = 5; AT7519, n = 5; and NVP-2, n = 2), those with quiescent disease (anti-TNF responsive; AT7519, n = 2; NVP-2, n = 2), and those not on anti-TNF therapy (flavopiridol, n = 8; AT7519, n = 14; and NVP-2, n = 6) at the time of endoscopy. Mean ± standard error of the mean. Two-way analysis of variance. *D*, RT-qPCR measuring *TNF, IFNG, IL17A,* and *IL22* transcripts following 3-hour treatment with 4 μM AT7519 or 1 μM NVP-2. Mean ± standard error of the mean; *TNF, IFNG,* and *IL17A,* n = 7; *IL22* n=4. Mann-Whitney *U* test.

We next turned our attention to the effects of CDK9 inhibition on pro-inflammatory cytokine production in LPMCs, measuring both Th1 and Th17 cytokines. We found that CDK9 inhibition led to a reduction in both Th1 (IFN-γ and TNF-α) and Th17 (IL-17A and IL-22) cytokines (Figure 4, *A–B*). Although all 3 compounds suppressed IFN-γ to similar levels, flavopiridol had a more moderate effect on TNF-α, whereas NVP-2 had no effect on this cytokine. These results echoed, albeit more strongly, experiments with PB CD4^+^ T cells that showed a more limited effect of NVP-2 on TNF-α production (Figure 1, *E*). These data were then stratified according to whether patients were receiving anti-TNF therapy and their disease activity. These analyses revealed no difference in CDK9i-mediated cytokine suppression in LPMCs isolated from patients who were anti-TNF resistant, anti-TNF responsive, or anti-TNF naïve (Figure 4, *C*). Finally, we used reverse transcription quantitative polymerase chain reaction (RT-qPCR) to examine whether these effects on intracellular cytokine expression were also observed at the transcriptional level. We observed a significant reduction in *IFNG* and *IL17A* mRNA levels in LPMCs following 3 hours of CDK9 inhibition (Figure 4, *D*). Additionally, AT7519 reduced *TNF* and *IL22* transcript levels, with NVP-2 exhibiting less of an effect on these cytokines. We conclude that Th1 and Th17 cytokine gene transcription is suppressed by CDK9 inhibition, with the more specific CDK9i NVP-2 having a more targeted effect. Furthermore, CDK9i were equally effective at suppressing Th1 and Th17 cytokines in lymphocytes isolated from patients who were resistant to anti-TNF therapy as those who were not.

### Restimulated Colonic CD4^+^ T Cells Possess a Memory Phenotype and are Highly Responsive to CDK9 Inhibition

We hypothesized that the reason why NVP-2-mediated TNF-α suppression was more effective in PB CD4^+^ T cells than LPMCs might be due to differences in cell activation. Although LPMCs underwent primary stimulation with PMA/I immediately after harvest, PB CD4^+^ T cells were activated with anti-CD3 and anti-CD28 for 72 hours before being restimulated with PMA/I. It is recognised that restimulated cells exhibit enhanced activation of signaling pathways downstream of the TCR, which prolongs transcriptional activity, amplifying downstream gene expression.^31^ Additionally, TCR activation primes gene promoters and enhancers for activation.^32^ It is therefore plausible that the primed state of genes renders restimulated T cells more susceptible to CDK9 inhibition.

**Figure 5:**
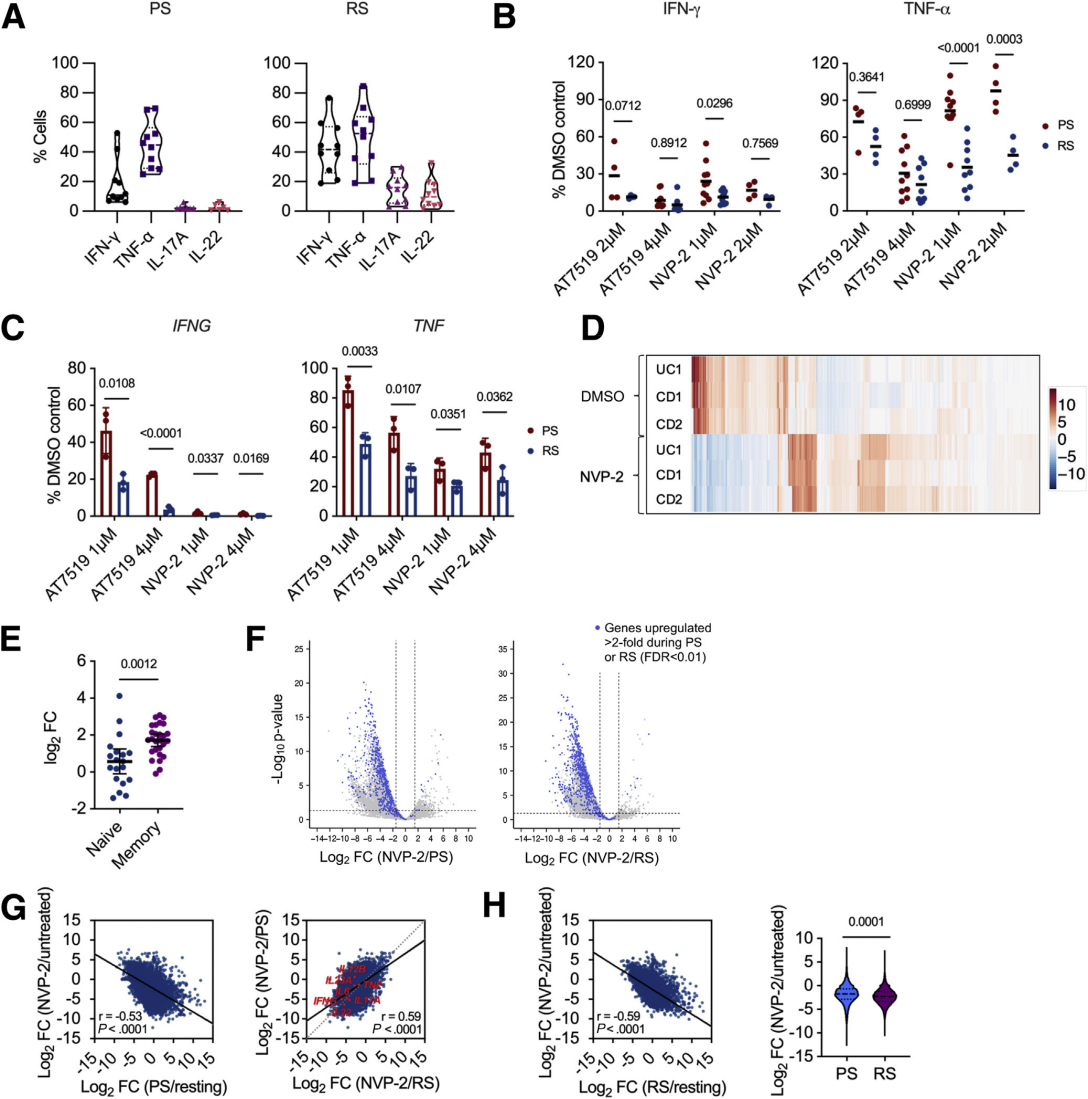
**Restimulated colonic CD4**^**+**^**T cells are highly responsive to CDK9 inhibition.***A*, Proportions of purified colonic CD4^+^ T cells positive for intracellular cytokines following primary stimulation (PS) or restimulation (RS). Median ± interquartile range; PS – IFN-γ & TNF-α (n = 10), IL-17A (n = 7), IL-22 (n = 6). RS - IFN-γ, TNF-α, IL-17A, IL-22 (n = 10). *B*, Proportions of purified colonic CD4^+^ T cells positive for intracellular cytokines following treatment with CDK9 inhibitors during PS or RS relative to untreated (DMSO control). 2μM AT7519 (AT) (n = 4), 4 μM AT7519 (AT) (n = 10), 1 μM NVP-2 (n = 10), 2 μM NVP-2 (n = 4). Two-way analysis of variance with the Šidák multiple comparisons test. *C*, RT-qPCR measuring *IFNG* and *TNF* expression in colonic CD4^+^ T cells following 3-hour treatment with AT7519 or NVP-2 during PS or RS. Mean ± standard error of the mean; n = 3. Unpaired 1-tailed Student *t* test. *D*, Heat map depicting changes in transcript levels for the top 500 genes ranked by inter-sample variability in NVP-2 treated and untreated restimulated colonic CD4^+^ T cells purified from 1 patient with UC and 2 patients with CD. *E*, Differential expression of genes specific to naïve (n = 19) or memory (n = 27) phenotypes in colonic CD4^+^ T cells following 72-hour activation with anti-CD3 and anti-CD28. Mean ± standard error of the mean. Mann-Whitney *U* test. *F*, Volcano plots of differential gene expression in cells treated with NVP-2 during PS or RS compared with cells left untreated during PS or RS. *Blue dots* represent genes upregulated (log_2_ fold-change ≥2; FDR <0.01) following PS or RS of untreated cells. *G*, Correlation between the degree of gene activation during PS and RS of untreated cells and the degree of gene repression by NVP-2 during PS and RS. Pearson correlation with *t* test. *H*, Degree of transcriptional repression caused by treatment of colonic CD4^+^ T cells with NVP-2 during PS compared with treatment during RS. Pearson correlation and *t* test (*left*) and violin plot and Mann-Whitney *U* test (*right*).

To test this hypothesis, we sought to evaluate the phenotypic and functional differences in primary stimulated and restimulated colonic CD4^+^ T cells. LPMCs from patients with IBD were harvested, and CD4^+^ T cells were isolated by fluorescence-activated cell sorting (FACS) or positive magnetic-activated cell separation (MACS). Colonic CD4^+^ T cells were then treated with AT7519 or NVP-2 during primary stimulation (PMA/I for 3 hours) or restimulation (anti-CD3 and anti-CD28 for 72 hours, rested for 48 hours, restimulated with PMA/I for 3 hours) and analyzed by flow cytometry and RT-qPCR. As expected, a greater proportion of restimulated colonic CD4^+^ T cells produced Th1 and Th17 cytokines when compared with primary stimulated cells (Figure 5, *A*). Notably, restimulated colonic CD4^+^ T cells were more sensitive to CDK9 inhibition, with greater reductions in IFN-γ and TNF-α production following culture with NVP-2 compared with primary stimulated cells (Figure 5, *B*). These findings were supported by RT-qPCR, which revealed a significant reduction in *IFNG* and *TNF* transcripts in restimulated cells compared with primary stimulated cells following CDK9 inhibition (Figure 5, *C*). We conclude that restimulated CD4^+^ T cells are more sensitive to CDK9 inhibition than their primary stimulated counterparts.

We sought to gain a more complete understanding of the effects of CDK9 inhibition on gene expression in primary stimulated and restimulated colonic CD4^+^ T cells. We purified colonic CD4^+^ T cells from 3 patients with IBD (2 with CD and 1 with UC) with active colonic inflammation, treated these with NVP-2 during primary stimulation or restimulation as before, and performed RNA-seq. First, we characterised the transcriptional signature of NVP-2-treated and untreated samples ranked by inter-sample variability. CDK9 inhibition had a consistent effect on gene expression in T cells derived from patients with both CD and UC (Figure 5, *D*). Using previously defined gene sets associated with naïve and memory CD4^+^ T cells,^33^ we identified that restimulation was associated with significant upregulation of memory-phenotype transcripts (Figure 5, *E*). Next, measuring the effect of CDK9 inhibition, we observed a global reduction in RNA pol II-transcribed RNAs in both primary stimulated and restimulated cells. However, although the effect was global, genes induced upon cell stimulation were particularly sensitive to CDK9 inhibition (Figure 5, *F*), with the degree of repression correlating with the intensity of transcriptional activation (Figure 5, *G*). Consistent with our findings for *IFNG* and *TNF*, this genome-scale analysis confirmed more potent transcriptional repression of genes in restimulated versus primary stimulated cells (Figure 5, *H*). We conclude that CDK9 inhibition effectively represses gene induction during primary and secondary stimulation of colonic CD4^+^ T cells, with a greater effect on restimulated, memory-phenotype cells.

### Genes Repressed by CDK9 Inhibition are Occupied by T-bet and P-TEFb

**Figure 6:**
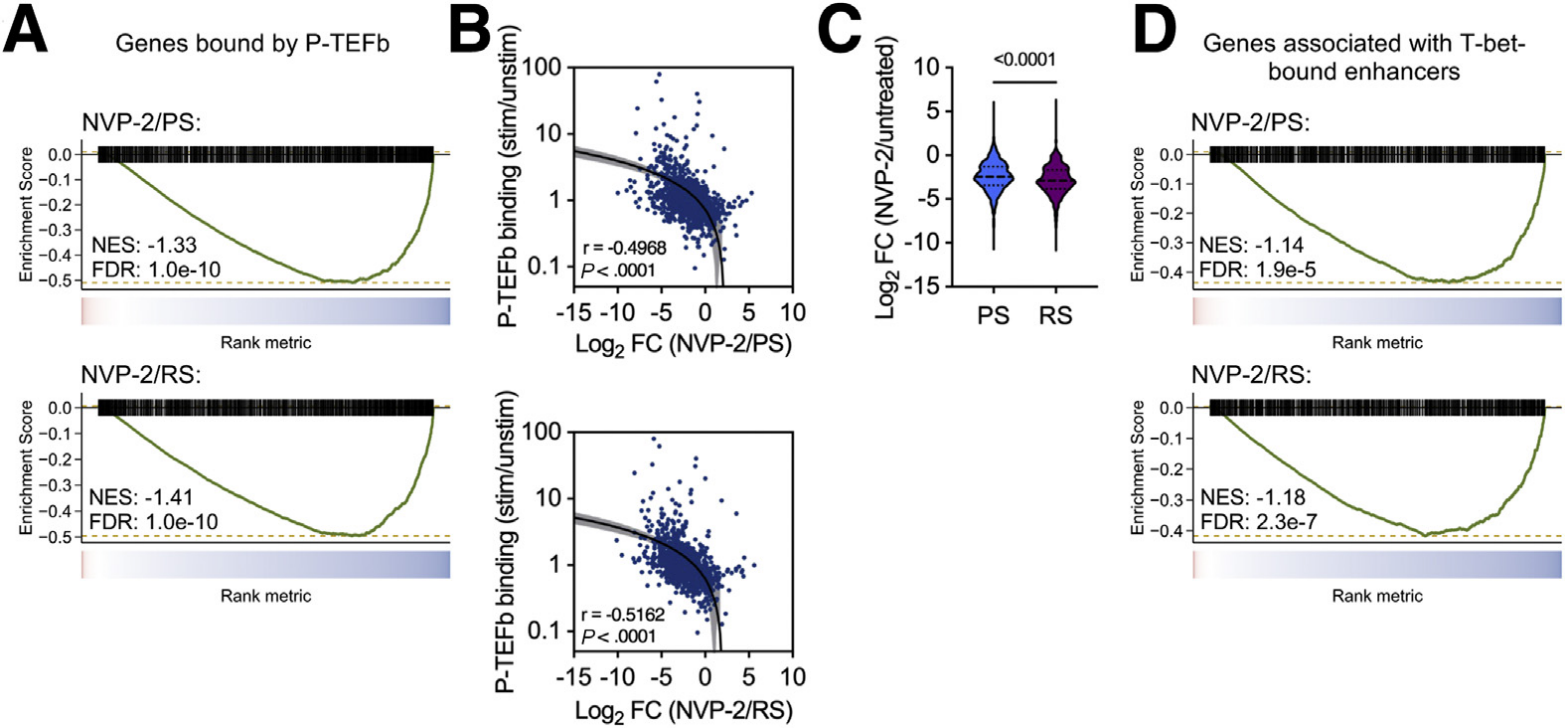
**Genes bound by P-TEFb and T-bet are more susceptible to CDK9 inhibition.***A*, GSEA of genes bound by P-TEFb in Th1 cells compared with changes in gene expression caused by NVP-2 treatment during primary stimulation (PS) or restimulation (RS) of colonic CD4^+^ T cells. *B*, Correlation between the change in P-TEFb occupancy upon cell stimulation and change in gene expression following NVP-2 treatment during PS and RS. n = 1727. Spearman correlation. *C*, Change in expression of P-TEFb bound genes following NVP-2 treatment during PS compared with RS. Median ± interquartile range, n = 936. Mann-Whitney *U* test. *D*, GSEA of genes associated with T-bet-bound enhancers compared with changes in gene expression caused by NVP-2 treatment during PS or RS of colonic CD4^+^ T cells.

Having established that CDK9 inhibition represses genes induced upon activation, we sought to determine whether this was related to the regulation of these genes by P-TEFb and T-bet. To investigate the association between sensitivity to CDK9 inhibition and P-TEFb occupancy, we identified genes bound by P-TEFb from existing ChIP-seq data^23^ and performed gene set enrichment analysis (GSEA). This revealed significant enrichment of genes occupied by P-TEFb among the genes repressed upon CDK9 inhibition (Figure 6, *A*). Next, we quantified the change in P-TEFb occupancy at transcription start sites (TSSs) upon stimulation and determined whether this correlated with transcriptional repression. Genes with increased recruitment of P-TEFb upon activation were more potently repressed following CDK9 inhibition, with the greatest effect observed in restimulated cells, consistent with our other findings (Figure 6, *B–C*). To evaluate whether T-bet target genes were more readily repressed following CDK9 inhibition, we utilised existing T-bet ChIP-seq data^23^ and performed GSEA as before. This demonstrated that genes associated with a T-bet-bound enhancer were significantly over-represented among the genes that were repressed by CDK9 inhibition (Figure 6, *D*). Collectively, these findings indicate that CDK9 inhibition preferentially represses T-bet target genes to which P-TEFb is recruited upon T cell activation.

### CDK9i-repressed Genes are highly Expressed in Anti-TNF-resistant IBD and Predict Response to Therapy

**Figure 7:**
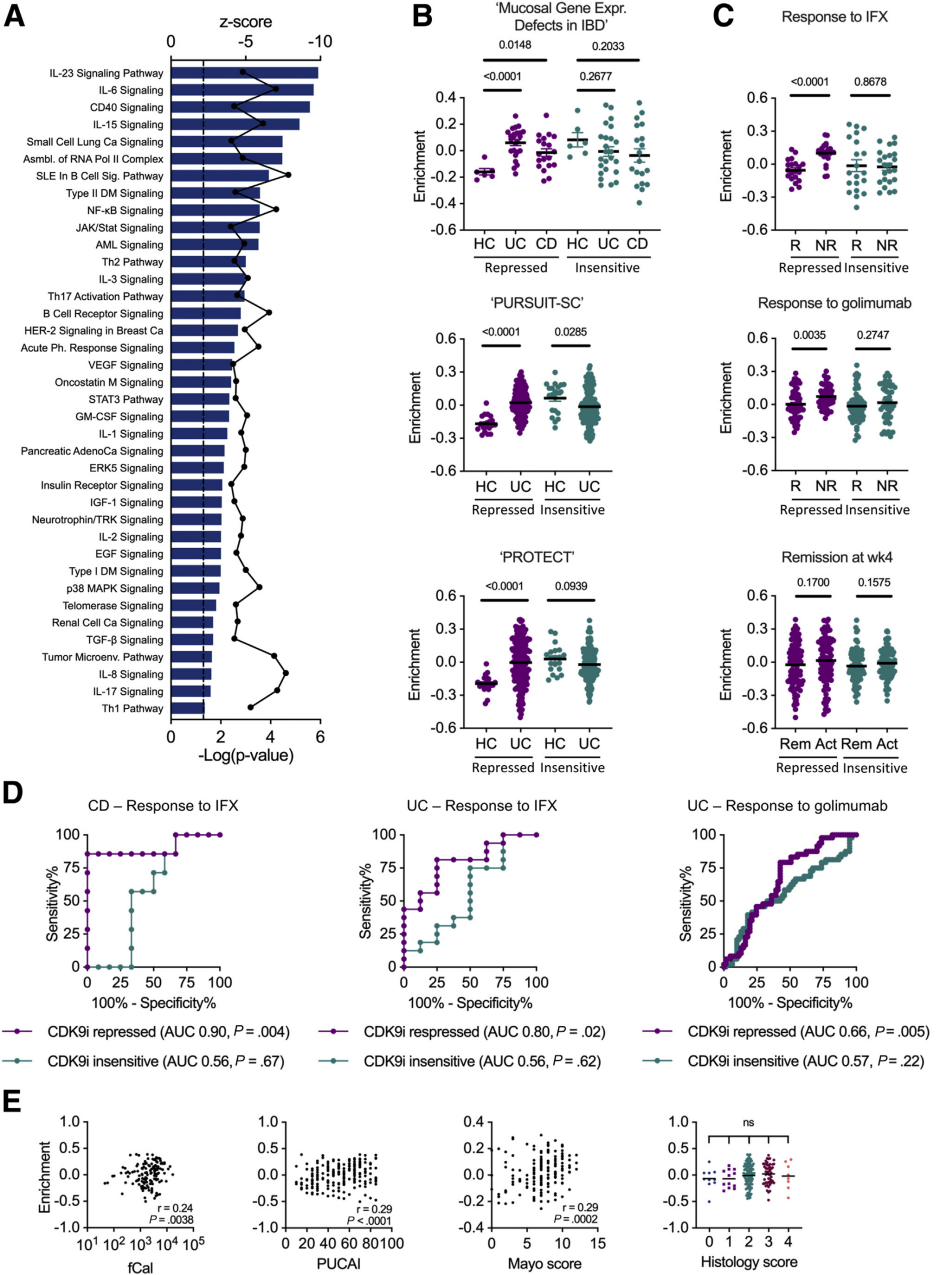
**CDK9i-repressed transcripts are enriched in anti-TNF resistant IBD.***A*, Ingenuity pathway analysis showing enrichment of genes repressed by NVP-2 (defined as log_2_ FC <−2 and FDR <0.01 in NVP-2 vs untreated restimulation [RS] cells) in inflammatory signaling and cancer pathways. *Bars* indicate −log_10_ (*P*-value), and *dots* indicate z-score. *B*, GSVA of NVP-2-repressed genes and genes insensitive to NVP-2 (defined as log_2_ FC = 0 in NVP-2/RS) in colonic CD and UC compared with healthy controls (HCs). Results for 3 independent IBD datasets (Mucosal Gene Expression Defects in IBD [GSE16879], PURSUIT-SC [GSE92415], and PROTECT [GSE109142]). Unpaired Student *t* test. *C*, GSVA of NVP-2-repressed and insensitive genes in anti-TNF responders (R) vs non-responders (NR), and remission (Rem) or active disease (Act) at week 4. Results for 3 independent IBD datasets outlined above. Unpaired Student *t* test. *D*, Receiver operator characteristic analysis of NVP-2-repressed genes and genes insensitive to NVP-2 in UC and colonic CD, distinguishing infliximab responders and non-responders in the Mucosal Gene Expression Defects in IBD data set and golimumab responders and non-responders in the PURSUIT-SC data set. *E*, Correlation of enrichment score of NVP-2-repressed genes with clinical parameters including fecal calprotectin (fCal), PUCAI, Mayo score, and histology score. Spearman correlation and *t* test.

We sought to understand the biological processes impacted by CDK9 inhibition. Ingenuity pathway analysis (IPA) of transcripts downregulated upon CDK9 inhibition revealed significant repression of immune pathways relevant to IBD and other inflammatory disorders including Th1, Th2, and Th17 pathways, and IL-6 and IL-23 signaling (Figure 7, *A*). Next, we probed the clinical relevance of CDK9i-repressed genes (defined as the top 250 genes repressed by NVP-2 with false discovery rate [FDR] <0.01) using gene expression data from colonic biopsies from an independent cohort of patients with IBD taken pre- and post-anti-TNF (infliximab) therapy (Mucosal Gene Expression Defects in IBD; ClinicalTrials.gov identifier: NCT00639821).^34^ Gene set variation analysis (GSVA) demonstrated significant enrichment of CDK9i-repressed transcripts in both UC and CD over healthy controls (Figure 7, *B*). We proceeded to analyze whether these transcripts were associated with responsiveness to anti-TNF therapy. Strikingly, our analysis revealed that CDK9i-repressed transcripts were significantly enriched in non-responders, suggesting that CDK9 inhibition may repress pro-inflammatory genes that are resistant to anti-TNF therapy (Figure 7, *C*). These findings were replicated in colonic biopsies sampled from patients with UC who participated in the PURSUIT-SC induction study (multicenter, randomized, double-blind, placebo-controlled trial of the anti-TNF-α antibody, golimumab, in moderate-severe UC; ClinicalTrials.gov identifier: NCT00487539)^8^ (Figure 7, *B*). Analysis of gene expression microarray data demonstrated significant enrichment of CDK9i-repressed transcripts in patients with UC who were non-responders to golimumab (Figure 7, *C*). This effect was specific for repressed transcripts and was not observed for transcripts that were insensitive to CDK9 inhibition (Figure 7, *B–C*). There was also enrichment of CDK9i-repressed genes in a pediatric UC cohort from the PROTECT study (Predicting Response to Standardised Paediatric Colitis Therapy; ClinicalTrials.gov identifier: NCT01536535).^35^ Interestingly, there was no enrichment of transcripts in children with UC who were resistant to conventional therapy with 5-aminosalicylic acid or corticosteroids, suggesting that CDK9 inhibition may be targeting a gene set specific for anti-TNF non-response. We therefore reasoned that the expression profile of CDK9i-repressed genes may predict response to anti-TNF therapy. Receiver operator characteristic analysis demonstrated that the transcriptional signature of CDK9i-repressed genes was a significant discriminatory factor in predicting response to anti-TNF therapy in CD and, to a lesser extent, UC (Figure 7, *D***)**. Finally, we asked whether enrichment for these transcripts correlated with clinical data that were available from the PURSUIT-SC and PROTECT studies. Only a weak correlation was observed between clinical severity scores (Mayo score and Paediatric UC Activity Index [PUCAI]), fecal calprotectin, and enrichment for CDK9i-repressed genes (Figure 7, *E*). Taken together, these results suggest that the CDK9i transcriptional signature may function as an independent predictor of response to anti-TNF therapy supplementary to existing clinical indices.

## Discussion

With the burden of IBD remaining significant despite the advent of several new treatments, including anti-TNF therapy, there is a pressing need to develop therapeutic options for those with resistant disease.^36^ CDK9 is emerging as an attractive target in both autoimmunity and oncology owing to its function in transcriptional elongation as part of P-TEFb. We have previously demonstrated that Th1 gene transcriptional activation requires P-TEFb and blockade of this pathway abrogates Th1-mediated uveitis.^23^ In agreement with this, Chen et al went on to describe a dual inhibitor of Cdc7/CDK9 that potently suppressed T cell activation, proliferation, and effector function.^37^ Early CDK9i were hindered by their lack of specificity, with off-target effects leading to adverse reactions.^38^ The advent of highly selective CDK9i has yielded promising safety data in phase I and II clinical trials in oncology and raised expectations for the development of a well-tolerated anti-inflammatory/anti-tumor agent.^39^^,^^40^ To our knowledge, the potential efficacy of CDK9 inhibition in gastrointestinal inflammation has only been evaluated in chemically-induced colitis models. The oral GSK3-α/β and CDK9 inhibitor, ABC1183, led to a dramatic improvement in dextran sulfate sodium- and 2,4,6-trinitrobenzene sulfonic acid- induced colitis that was associated with suppression of TNF-α and IL-6, and no observed organ or hematological toxicity.^41^ In this study, we evaluated CDK9 inhibition in adaptive immune-mediated colitis and explored its effect on the transcriptional landscape and effector function of colonic CD4^+^ T cells from patients with IBD.

Transcriptional elongation is stimulated by CDK9-mediated phosphorylation of RNA pol II serine 2, which forms a checkpoint for transcriptional activation.^23^^,^^42^ In our model, CDK9 inhibition was associated with diminished RNA pol II S2P, indicating disruption of transcriptional elongation. This was accompanied by suppression of IFN-γ and TNF-α production by both mouse and human CD4^+^ T cells. CDK9 inhibition also reduced IFN-γ and TNF-α production in TCT colonic explants, suggesting that these small molecule inhibitors are readily absorbed into the intestinal mucosa where they directly suppress tissue resident immune cells.

In light of these observations, we extended our study to investigate the effect of systemic CDK9 inhibition in a preclinical model of IBD. TCT colitis is driven by aberrant Th1 and Th17 effector responses resulting in transmural colonic inflammation. Systemic CDK9 inhibition resulted in an improved colitis score associated with diminished Th1 and Th17 cytokine production by colonic CD4^+^ T cells. This immunosuppressive effect was confined to colonic T cells, suggesting that CDK9 inhibition may have the greatest effect on activated lymphocytes within sites of inflammation. In this model, the inhibition of T-bet-dependent genes was not associated with a switch to Th17 cell differentiation, as evidenced by the reduction in colonic IL-17A expression in treated mice. This was further supported by the IPA in human colonic CD4^+^ T cells demonstrating that CDK9 inhibition represses Th17 pathways. The improvement of colitis score was less marked with NVP-2 compared with flavopiridol. To our knowledge, this is the first time that NVP-2 has been tested in a disease model, and it is likely that the administered dose was at the lower end of the therapeutic window. Thus, further optimization of the NVP-2 dosing schedule is required.

In CD, colonic lymphocytes can exhibit phenotypic differences to their PB counterparts that confer resistance to anti-inflammatory signalling at mucosal surfaces.^43^ Accordingly, it was important to determine the effect of CDK9 inhibition on the function of colonic lymphocytes. Treatment of colonic LPMCs with CDK9i repressed genes encoding Th1 and Th17 cytokines but to a lesser extent than in PB CD4^+^ T cells. Differences in cytokine repression between PB and colonic lymphocytes were attributable to differences in cellular activation and therefore immunological memory. We demonstrated that restimulated colonic CD4^+^ T cells readily expressed memory phenotype markers, produced more Th1 and Th17 cytokines, and were more sensitive to CDK9 inhibition compared with primary stimulated cells. This is particularly relevant as memory T cells are known to accumulate in the intestinal mucosa of patients with IBD.^44^^,^^45^

The degree to which a gene was repressed following CDK9 inhibition correlated with the extent to which the gene was induced during T cell activation. To gain further mechanistic insight, P-TEFb occupancy at TSSs was quantified by ChIP-seq and compared with changes in gene expression. Genes bound by P-TEFb were significantly enriched among CDK9i-repressed transcripts. Furthermore, CDK9 inhibition had its greatest repressive effect on genes to which P-TEFb was recruited upon T cell activation, suggesting that CDK9 inhibition primarily blocks gene induction rather than steady-state gene expression. We conclude that highly activated genes are more susceptible to CDK9 inhibition due to increased recruitment of P-TEFb upon T cell activation. This might explain why systemic CDK9 inhibition in the TCT model led to cytokine suppression in colonic T cells but not those in the mesenteric lymph nodes or spleen. We have previously reported that T-bet recruits P-TEFb to activate transcriptional elongation of Th1 genes.^23^ In agreement with this, genes associated with T-bet-bound enhancers were significantly enriched among CDK9i-repressed transcripts. Gene repression following CDK9 inhibition therefore exhibits some specificity for T-bet target genes, and more broadly for genes that are induced upon T cell activation by recruitment of P-TEFb.

The changes in gene expression caused by CDK9 inhibition were broadly similar for T cells from our cohort of 1 patient with UC and 2 patients with CD, and this may be explained by overlap between the immunopathological pathways driving intestinal inflammation in colonic CD and UC. CDK9i-repressed transcripts were enriched in numerous immune pathways implicated in IBD and auto-immunity. Using colonic transcriptomic data from 3 independent cohorts of patients with IBD (1 with CD and UC, 1 with UC, 1 with pediatric UC), we established that CDK9i-repressed transcripts were associated with IBD and more specifically with non-response to anti-TNF. Collectively, the enrichment of CDK9i-repressed genes in anti-TNF resistance, together with the ability of CDK9i to suppress cytokine production in LPMCs from anti-TNF non-responders, provides compelling evidence that CDK9 inhibition may have therapeutic potential in patients who have not responded to anti-TNF therapy.

The ability to predict disease course and response to therapy has great potential to transform the management of IBD, enabling a personalized approach to care. Conventional measures including clinical/endoscopic activity scores (Mayo, Crohn’s Disease Activity Index) and biochemical indices (C-reactive protein, fecal calprotectin) have proved useful for monitoring patients but remain inadequate for predicting response to therapy. Our analysis revealed that the gene expression profile of CDK9i-repressed transcripts was predictive of anti-TNF response in 2 independent cohorts of patients with IBD with the greatest effect observed for colonic CD. There was only weak correlation between enrichment for CDK9i-repressed transcripts and clinical indices commonly associated with disease activity (Mayo, PUCAI, fecal calprotectin), suggesting this transcriptional signature may function independently of conventional biomarkers. These findings raise the possibility of utilising this CDK9-dependent transcriptional signature as a predictive biomarker for anti-TNF response.

In summary, using an immune-mediated murine colitis model and colonic lymphocytes from patients with IBD, we demonstrate the therapeutic potential of inhibiting P-TEFb/CDK9, a transcriptional elongation factor downstream of T-bet. Mechanistically, CDK9 inhibition primarily represses genes induced by the recruitment of P-TEFb following T cell activation. CDK9i-repressed genes are implicated in multiple immune pathways and are associated with anti-TNF non-responsive IBD. This raises the prospect of CDK9 inhibition as a potential therapeutic option in anti-TNF resistant disease.

## Methods

### Study Approval

BALB/c *Rag2*^-/-^ and C57BL/6 WT mice were sourced commercially (Jackson Laboratories). Animal experiments were performed in accredited facilities in accordance with the UK Animals (Scientific Procedures) Act 1986 (Home Office License Number PPL: 70/7869 to September 2018; P9720273E from September 2018). Studies in human tissues received ethical approval from the London Dulwich Research Ethics Committee (REC reference 15/LO/1998). Written informed consent was received from all participants prior to inclusion in the study. Patient demographics are outlined in Table 1.

**Table 1:** Demographics of Patients With CD and UC

	Patients with CD	Patients with UC
Patients	71	56
Male/female	38/33	37/19
Mean age, *y*	36.9	44.7
Disease activity		
Quiescent/mild	30	24
Moderate/severe	41	32
Treatment		
Nil	12	5
Conventional therapy/steroids	17	32
Anti-TNF (infliximab, adalimumab)	34	9
Anti-integrin (vedolizumab)	5	5
Anti-IL12/23 p40 (ustekinumab)	3	1
Anti-JAK (tofacitinib)	0	1

CD, Crohn’s disease; IL, interleukin; JAK, Janus kinase; TNF-α, tumor necrosis factor-alpha; UC, ulcerative colitis.

### T cell Transfer Experiments

*Rag2*^*-/-*^ mice received intraperitoneal injection of 0.5 × 10^6^ syngeneic CD4^+^CD25^-^CD62L^+^CD44^low^ T cells. Mice developed clinical signs of colitis from day 28 to 32 following T cell transfer. Mice with clinical signs of disease received either 3 mg/kg flavopiridol, 1 mg/kg NVP-2, or DMSO (0.8% DMSO in 10% cyclodextrin) once daily. On day 6 of treatment, mice were sacrificed and assessed for severity of colitis with weights and biopsies taken from the distal colon.

### Murine Colonic LPMC Isolation

Mice were euthanised by cervical dislocation, and spleens, mesenteric lymph nodes, and colons were removed and placed in ice-cold phosphate buffered saline (PBS). Colons were opened longitudinally, rinsed thoroughly and cut into 1- to 2-mm pieces and washed in epithelial cell removal buffer (5 mM EDTA and 1 mM Hepes in Hank's balanced salt solution) in a shaking water bath at 37^o^C for 20 minutes. Tissue was vortexed vigorously and filtered through a 100-μM cell strainer and collected in complete RPMI (Gibco) containing 10% fetal calf serum (FCS), 0.25 mg/mL collagenase D (Roche), 1.5 mg/mL dispase II (Roche), and 0.01 μg/mL DNase (Roche) and put in a shaking water bath (300 rpm) at 37 ^o^C for 40 minutes. Solutions were then filtered through 100-μM cell strainers and washed with ice-cold PBS. Cells were then resuspended in 10 mL of the 40% fraction of a 40:80 Percoll gradient and placed on top of 5 mL of the 80% fraction in 15-mL tubes. Percoll density gradient separation was performed by centrifugation at 900 × g for 20 minutes at room temperature. The interphase containing LPMCs between 40% and 80% Percoll was collected, filtered, and prepared for use in further experiments.

### Histology

Murine colonic biopsies were fixed with 10% paraformaldehyde and embedded in paraffin blocks. Five-μM sections were stained with hematoxylin and eosin. Histological colitis scores were calculated in a blinded fashion as previously described.^46^

### Ex Vivo Organ Culture

Three-mm punch biopsies (Miltex) were used to acquire full thickness murine colonic specimens. Biopsies were cultured in 1 mL of complete media for 27 hours. Cytokine concentrations in culture supernatants were measured by ELISA (R&D Systems).

### Human Colonic LPMC Isolation

Patients with IBD were recruited to the study prior to endoscopy at Guy’s & St Thomas’ NHS Foundation Trust. Colonic biopsies were taken from areas of active inflammation or from the recto-sigmoid in cases with minimal inflammation at the time of endoscopy. Colonic biopsies were cultured on Cellfoam matrices in complete media (RPMI with 10% FCS) with antibiotics and IL-2 (0.1 IU/ml) for 48 h. Colonic LPMCs were then isolated from the supernatant ready for evaluation.^30^ Informed written consent was obtained in all cases.

### Cell Culture

#### Murine Th1 cell stimulation

Naïve CD4^+^ T cells were purified from C57BL/6 mice, activated with plate-bound anti-CD3 and anti-CD28 (2 μg/ml) under Th1-polarising conditions (complete RPMI [10% FCS, 100 U/mL penicillin, 100 μg/mL streptomycin, 2 mM L-glutamine, 0.1 mM non-essential amino acids, 10 mM HEPES, 50 μM 2-mercaptoethanol, 1 mM sodium pyruvate] containing IL-2 [1 IU/mL], anti-IL-4 [10 μg/mL], and IL-12 [10 ng/mL]) for 72 hours. Cells were then cultured in complete media with IL-2 (1 IU/mL) for an additional 4 days then re-activated with PMA (50 ng/mL) and PMA/I (1 μM) in the presence of CDK9i for 4 hours.

#### Human LPMC primary stimulation

Human LPMCs were cultured in complete media with PMA (50 ng/mL), ionomycin (1 μM), monensin (2 μM), brefeldin A (5 μg/mL), and CDK9i at the indicated doses for 3 hours at 37^o^C prior to FACS staining.

#### Human PB and colonic CD4^+^ T cell restimulation

Human peripheral blood mononuclear cells were isolated by density gradient centrifugation over lymphocyte separation media (LymphoPrep). LPMCs were extracted from colonic biopsies as previously described.^30^ CD4^+^ T cells were then isolated by FACS or positive MACS. CD4^+^ T cells were cultured in complete media containing IL-2 (1 IU/mL) on plate-bound anti-CD3 and anti-CD28 (2 μg/mL) for 72 hours. Cells were rested in complete media with IL-2 (1 IU/mL) for an additional 48 hours then restimulated with either PMA (50 ng/mL) and ionomycin (1 μM) or anti-CD3 and anti-CD28 microbeads (MACS GMP ExpAct Treg kit, 10 μl/10^6^ cells) in the presence of CDK9i for 3 hours. Cells were taken out of culture, and beads were removed prior to staining. For cytokine quantification by ELISA, cells were activated for 16 hours rather than 3 hours, then supernatant taken.

### Flow Cytometry

Cells were stimulated using the experimental protocols outlined above with the addition of monensin (2 μM) and brefeldin A (5 μg/mL). Surface staining antibodies were added following a live/dead fixable viability stain (eBioscience Fixable Viability Dye eFluor 780, Invitrogen FxCycle violet stain). For intracellular staining, cells were fixed and permeabilised using the Foxp3 fixation/permeabilization buffer kit (ThermoFisher) or 4% paraformaldehyde followed by 0.2% saponin buffer. Following staining, cells were washed and resuspended in FACS buffer (5% FCS and 2 mM EDTA in 1× PBS) and stored in the dark at 4 ^o^C awaiting acquisition. Samples were acquired using a BD LSRFortessa (BD Biosciences) flow cytometer. Data were recorded using BD FACSDiva 6.0 and analysed using FlowJo software (Treestar Inc). FACS antibodies were sourced from BioLegend unless otherwise stated. Antibodies for human proteins were as follows: CD8-BUV737 (SK1) (BD), CD4-BV785 (OKT4), CD4-FITC (OKT4) (eBioscience) CD3-PE-Cy7 (OKT3), CD45-AF700 (2D1), IFN-γ-BV605 (4S.B3), IFN-γ-APC (4S.B3), TNF-α-BUV395 (MAb11) (BD), IL17A-BV421 (BL168), IL22-APC (2G12A41), and IL10-PE (JES3-19F1). Antibodies for mouse proteins were as follows: CD45-BV510 (30-F11), CD3-Pac Blue (17A2), CD4-FITC (GK1.5), CD4-AF700 (RM4-5), CD25-APC (PC61), CD44-PE-Cy7 (IM7), CD62L-FITC (MEL-14), IL17A-AF700 (TC11-18H10.1), TNF-α-APC-Cy7 (MP6-XT22), IFN-γ-PE (XMG1.2), and IL22-APC (Poly5164).

### ELISA

Cytokine concentrations (IFN-γ and TNF-α) were measured in culture supernatants by ELISA (Biolegend).

### Real-time Quantitative PCR

Cells were lysed in RLT buffer (Qiagen), and RNA was extracted using RNeasy mini kit (Qiagen). Complementary DNA was generated with the RevertAid First Strand cDNA synthesis kit (Thermo Scientific). Multiplex qPCR was used to quantify mRNA in triplicate using TaqMan gene expression assays (Applied Biosystems: Human *TNF* (Hs00174128_m1), *IFNG* (Hs00989291_m1), *IL17A* (Hs00174383_m1), *IL22* (Hs01574154_m1), and *ACTB* (4326315E)). Gene expression was quantified relative to *ACTB* using the 2^-ΔCT^ method.

### RNA-seq

FACS-purified colonic CD4^+^ T cells were lysed in RLT buffer (Qiagen) and RNA extracted using RNeasy mini kit (Qiagen). Libraries were prepared from 10 ng total RNA using the NuGEN Ovation SoLo RNA-Seq Library Preparation kit and then sequenced on an Illumina HiSeq 3000 with 150 bp single-end reads. From sequenced raw reads, adapter sequences were trimmed using Trim Galore! and 3'-ends with low quality bases (Phred quality score < 20) were removed using FastQC. In addition, only concordantly and uniquely mapped read pairs were used for analysis. Kallisto^47^ was used for quantification of transcript-level abundances by mapping QC-filtered reads to all ENSEMBL transcripts of the GRCh38 reference genome. Gene expression levels were computed using tximport^48^ by summing up transcript level read counts for all transcript isoforms at the gene level. Lowly expressed genes (<6 counts in ≤2 samples) were removed from the count matrix before normalization between samples. Due to a repressive effect of NVP-2 on all pol II-transcribed genes, the data were normalized to the average of the following RNA pol I and pol III transcripts whose expression is not affected by P-TEFb inhibition: *RNA5S12, RNA5S11, RNA5S3, RNA5S1, RNA5S5, RNA5S10, RNA5S8, RNA5S17, RNA5S4, RNA5S6, RNA5S9, RNA5S14, RNA5S2, RNU6-7, RNA5S15, RN7SK, RNA5S16, RNU6-8, RNA5S7, RNA5S13, RNU6-1, RNU6-2, RNU6-9, RMRP, RN7SL2, RNA5-8SN3, RN7SL1, RPPH1, RNA5-8SN1, RNA5-8SN2,* and *RN7SL3*. Normalized read counts were log-transformed and differentially expressed genes between 2 sample groups were identified using a negative binomial generalized linear model for unpaired comparisons as implemented in the DESeq2^49^ R package. *P*-values were corrected for multiple comparisons using the Benjamini-Hochberg method. All genes with adjusted *P*-value of < .05 were considered differentially expressed.

### ChIP-seq Data Analysis

Significant P-TEFb and T-bet peaks were identified from previously published ChIP-seq data (GSE62486^23^) with MACS version 1.4 using a *P*-value threshold of 10^-7^. As outlined by Hertweck et al, ChIP-seq data were derived from human naïve T cells that were isolated and cultured under Th1 polarizing conditions for 13 days.^23^ ClosestBed was used to allocate peaks to the most nearby gene defined by the shortest distance to a RefSeq gene TSSs. Genes with at least one P-TEFb peak within 2 kb of their TSSs were defined as P-TEFb bound genes, and T-bet enhancer-associated genes were defined as genes with at least one T-bet binding site ≥2kb from their TSSs.

P-TEFb occupancy at TSSs was quantified by enumerating the reads using featureCounts (http://bioinf.wehi.edu.au/featureCounts/)^50^ and converting to reads per kilobase per million using the size of the TSS region and the total number of aligned reads in that sample. The number of reads in the input sample were then subtracted.

### Gene Set Enrichment Analysis

Enrichment of P-TEFb bound or T-bet enhancer-associated gene sets was computed using the fgsea R package^51^ using gene lists ranked by −log_10_ *P*-value divided by the sign of the fold change for gene lists. The gene set was limited to genes expressed in CD4^+^ T cells by filtering out genes with fragments per kilobase million <1 in the untreated and treatment conditions for each stimulation.

To test the activation of the NVP-2 inhibited transcriptional programme in the context of IBD we used GSVA^52^ to probe previously reposited datasets of whole transcriptional profiles of patients with IBD (GSE16879,^34^ GSE92415,^53^ and GSE109142^35^). In all 3 studies, GSVA was performed on transcriptomic data derived from pre-treatment samples. The NVP-2 transcriptional signature (gene set) was defined as the 250 genes most highly repressed by NVP-2 (ranked by fold change, FDR <0.01). A control gene set of 110 genes, defined as unresponsive to NVP-2 (log_2_ fold change = 0), were used for comparison. Enrichment scores were generated using the gsva option of the GSVA R package using default settings. The enrichment score varies between −1 to +1, with results >0 suggesting that the gene set is enriched (active/upregulated) and results <0 suggesting that the gene set is inhibited (downregulated).

### Ingenuity Pathway Analysis

To evaluate the biological systems affected by NVP-2, IPA was undertaken using IPA (QIAGEN, version 01-13). Analysis was performed on genes repressed by NVP-2 (defined as log_2_ FC <-2 and FDR <0.01 in the NVP-2/RS gene set).

#### Accession numbers

The accession number for the RNA-seq data reported in this study is GEO: GSE172372.

## Immunoblotting

Human PB CD4^+^ T cells were isolated and cultured on plate-bound anti-CD3 and anti-CD28 for 72 hours, as described (see ‘Human PB and colonic CD4^+^ T cell restimulation’). Cells were then rested for 48 hours and restimulated with PMA and ionomycin in the presence of CDK9i for 3 hours. Samples were washed with 1× PBS and lysed with 1 mL of TOPEX+ buffer (300 mM NaCl, 50 mM Tris-HCl pH 7.5, 0.5% Triton X-100, 1% SDS, 1 mM DTT, 1X Complete EDTA-free Protease Inhibitor Cocktail [Roche Applied Science], 1:49 Phosphatase Inhibitor Cocktail I [Abcam], 33.33 U/mL benzonase [Merck], 5 mM NaF, 0.2 mM sodium orthovanadate, and 10 mM β-glycerophosphate, disodium salt, pentahydrate). Total protein was quantified using the BCA Protein Assay Kit (Pierce). Twenty μg total protein was separated by sodium dodecyl sulfate–polyacrylamide gel electrophoresis. After transfer, membranes were blocked in PBS-Tween 5% milk for 1 hours, and proteins were detected using anti-RNA pol II CTD (8WG16, ab817 Abcam), anti-RNA pol II Ser2P (ab5095 Abcam), β-actin (4967S Cell Signalling Technology).

## Statistics

Results are expressed as mean ± standard error of the mean or median ± interquartile range unless otherwise stated. Data were checked for normality and parametric or non-parametric statistical analyses were performed as appropriate. Data were analyzed using the Student *t* test, Mann-Whitney *U* test, 2-way analysis of variance, and Spearman correlation as indicated, using GraphPad Prism 8.0 (GraphPad Inc).
